# Corrigendum to “Dexmedetomidine Postconditioning Alleviates Hypoxia/Reoxygenation Injury in Senescent Myocardial Cells by Regulating lncRNA H19 and m6A Modification”

**DOI:** 10.1155/2020/7059751

**Published:** 2020-08-24

**Authors:** Xuan Zhang, Qiang Fu, Longhe Xu, Yitian Yang, Weixing Zhao, Yunliang Zhang, Weidong Mi, Hao Li

**Affiliations:** ^1^Anesthesia and Operation Centre, Chinese PLA General Hospital, Beijing 100853, China; ^2^Department of Anesthesiology, Tianjin Medical University Cancer Institute and Hospital, Tianjin 300060, China

In the article titled “Dexmedetomidine Postconditioning Alleviates Hypoxia/Reoxygenation Injury in Senescent Myocardial Cells by Regulating lncRNA H19 and m6A Modification” [1], the author order in the author list was incorrect, where

“Xuan Zhang^1,2^, Qiang Fu^1^, Longhe Xu^1^, Yitian Yang^1^, Weixing Zhao^1^, Yunliang Zhang^1^, Hao Li^1^, and Weidong Mi^1^”should have read as follows:

“Xuan Zhang^1,2^, Qiang Fu^1^, Longhe Xu^1^, Yitian Yang^1^, Weixing Zhao^1^, Yunliang Zhang^1^, Weidong Mi^1^, and Hao Li^1^”

The correct author order is also shown above in the author information.
